# Comparing Substance Use and School-Based Stressors Among Black and Latinx Transgender Youth and Peers With Shared Minoritized Identities

**DOI:** 10.1016/j.jadohealth.2022.08.029

**Published:** 2022-10-09

**Authors:** Stanley R. Vance, Cherrie B. Boyer, David V. Glidden, Jae Sevelius

**Affiliations:** a Division of Adolescent and Young Adult Medicine, Department of Pediatrics, University of California, San Francisco, San Francisco, California; b Division of Adolescent and Young Adult Medicine, Department of Pediatrics, University of California, San Francisco, San Francisco, California; c Department of Epidemiology and Biostatistics, University of California, San Francisco, San Francisco, California; d Department of Medicine, Center for AIDS Prevention Studies, University of California, San Francisco, San Francisco, California

**Keywords:** Transgender youth, Schools, Minority stress, Bullying

## Abstract

**Purpose:**

The aim of this study is to compare substance use and school-based stressors among Black and Latinx transgender youth (trans BLY), White transgender youth (trans WY), and Black and Latinx cisgender youth (cis BLY) and identify associations between substance use and stressors among trans BLY.

**Methods:**

We analyzed 2015—2017 Biennial California Healthy Kids Survey data with a weighted sample of the state’s secondary school population. The analytic sample included 9th and 11th grade trans BLY, trans WY, and cis BLY. Past 30-day and lifetime substance use (cigarettes, e-cigarettes/vaping, marijuana, and alcohol) and school-based stressors (victimization, race-, gender-, and sexuality-based harassment) were compared between cohorts via logistic regression. For trans BLY, associations between substance use and stressors were assessed via logistic regression.

**Results:**

The analytic sample (n = 19,780) included 252 trans BLY, 104 trans WY, and 19,424 cis BLY. Among trans BLY, estimated prevalence of 30-day (and lifetime) use of cigarettes, e-cigarette/vaping, marijuana, and alcohol were 13% (23%), 19% (39%), 27% (42%), and 29% (48%), respectively. Trans BLY had similar odds of 30-day and lifetime use of all substances compared to trans WY but higher odds of use compared to cis BLY. For trans BLY, race- and gender-based harassment and higher victimization levels were associated with higher odds of 30-day and lifetime use of all substances. Sexuality-based harassment was associated with higher odds of 30-day and lifetime marijuana and alcohol use.

**Discussion:**

Trans BLY have high prevalence of substance use, comparable with trans WY but higher than cis BLY. Substance use among trans BLY is associated with school-based stressors.

Despite the decline in adolescent use of substances such as cigarettes and alcohol in the last two decades [1], substance use continues to be a prevalent public health problem affecting adolescents in the United States [2]. The rapid expansion of recreational marijuana legalization across the United States and vaping device use are recent factors that may affect newer trends in adolescent substance use [3]. The potential negative effects of substance use on adolescent neurocognitive function have been well-documented [4,5]. For example, marijuana use and alcohol use have been associated with decreased visual-spatial functioning, attention, memory, and psychomotor speed in adolescents [6–8]. Furthermore, substance use during adolescence is associated with increased risk of substance abuse in adulthood [9].

Studies have suggested that minoritized youth have higher prevalence of substance use when compared to peers. Specifically, transgender and gender-diverse youth—youth whose gender identity does not align with societal expectations ascribed to their sex designated at birth—have higher rates of substance use compared to their cisgender (non—transgender/gender diverse) counterparts [10–12]. Similarly, while frequent marijuana use is decreasing among White youth, frequent marijuana use has increased among both Black and Latinx youth [13,14]. Divergent rates and patterns in substance use point to the need to compare youth subgroups by race, ethnicity, and gender identity to examine substance use disparities among minoritized youth populations. Moreover, specifically examining substance use among Black and Latinx transgender youth (trans BLY), who have minoritized experiences based on race, ethnicity, and gender, is critical to determine their specific risk for substance use.

School is one of the most important socioecological domains for youth; thus, exploring factors that influence substance use among trans BLY in this setting is particularly salient. School is a key environment for engagement with peers, and peer influence is strongly associated with adolescent substance use [15,16]. Negative peer experiences within schools are associated with increased substance use for youth among minoritized students. For example, among gender-diverse youth, school-based victimization is a risk factor for substance use [10]. One study found that gender nonconformity increased risk of substance use, particularly among students who were assigned male at birth, and experiences of school-based victimization mediated this increased risk [17]. Similarly, among racial and ethnic minoritized youth, school-based racial discrimination increases risk of substance use [18]. Understanding school-based factors that impact risk of substance use among trans BLY is particularly relevant for developing culturally informed approaches to support them in reducing substance use.

The minority stress theory posits that minoritized individuals experience social stressors unique to their minoritization. When these stressors are internalized, they increase the vulnerability of minoritized individuals to poor health outcomes and behaviors that may increase their risk for such outcomes [19,20]. This theory initially focused on sexual minority individuals but later was expanded to apply to experiences of gender-diverse individuals with the gender minority stress framework [21,22]. This framework helps to conceptualize how trans BLY may be vulnerable to substance use due to social stressors related to minoritized experiences of being both gender diverse and racial and ethnic minorities. There is a dearth of data focused specifically on substance use among trans BLY. Prior studies of substance use among gender-diverse youth populations have not explored differences between White transgender youth (trans WY) and trans BLY [10–12,23]. To our knowledge, there have been no studies that have compared differences between trans BLY and Black and Latinx cisgender youth (cis BLY). Comparing trans BLY to two sets of peers—trans WY which they share the experiences of gender minoritization and cis BLY which they share experiences of racial and ethnic minoritization—could provide insight to the unique risks that trans BLY have for substance use and factors that may be associated with such risk. Therefore, it is critical that substance use and the antecedent social stressors among trans BLY are carefully examined to inform the development and implementation of culturally appropriate prevention interventions.

Exploring associations among substance use and school-based social stressors is critical in understanding health disparities experienced by trans BLY. Utilizing a large, statewide school-based survey sample representative of the California high school population, we derived three peer groups by combining measures of race, ethnicity, and gender identity: trans BLY, trans WY, and cis BLY. Our study had three specific aims. First, to explore differences in past 30-day and lifetime use of cigarettes, e-cigarettes/vaping, marijuana, and alcohol, we compare trans BLY to two groups of youth with whom they share at least one minoritized identity. That is, trans BLY was compared with trans WY and cis BLY. Second, we compare peer groups to assess differences in school-based stressors, including victimization, sexuality-based harassment, gender-based harassment, and race-based harassment. Finally, for trans BLY, we examine associations between school-based stressors and substance use. Informed by the gender minority stress framework, we hypothesize that trans BLY will report higher rates of substance use compared with trans WY and cis BLY, that trans BLY will report higher rates of school-based stressors compared with trans WY and cis BLY, and that among trans BLY, substance use will be associated with school-based stressors.

## Methods

### Data source

Survey data were collected from 9th and 11th grade students who participated in the Biennial State California Healthy Kid Survey (CHKS) administered from Fall 2015 to Spring 2017. The California Healthy Kid Survey is administered biennially by WestEd with support from the California Department of Education and California Department of Health Care Services. The anonymous survey is a modular, self-reported assessment that comprehensively attains youth health risk and resilience data. One hundred twenty California schools were randomly selected to participate. Opt-out parental consent and student assent were required for survey participation in compliance with state law [24]. The average response rate across participating schools was 70%. Of all respondents, 1.7% were excluded due to missing data in required survey modules, and an additional 1.1% were excluded due to questionable response validity. Student data were weighted to more closely reflect the demographics of the California secondary school student population. The final sample included 45,269 students. The California Office of Statewide Health Planning and Development Committee for the Protection of Human Subjects approved survey implementation. The University of California, San Francisco, Institutional Review Board approved this study.

### Measures

#### Sociodemographic measures.

One item assessed both gender identity and sexual identity: “Which of the following best describes you?” (Mark all that apply: Heterosexual/straight, gay/lesbian, bisexual, transgender, not sure, decline to respond). Participants were categorized as transgender for those who marked “transgender” and as cisgender for those who did not. The survey queried students on their race (“What is your race?”) and whether they were of Hispanic/Latino origin (yes/no). Three mutually exclusive race and ethnicity groups were created: Hispanic/Latinx, non-Hispanic/Latinx Black, and non-Hispanic/Latinx White. Peer groups combining race, ethnicity, and gender identity were created: trans BLY (transgender students who are non-Hispanic/Latinx Black or Hispanic/Latinx), trans WY (transgender students who are non-Hispanic/Latinx White), and cis BLY (students who are non-Hispanic/Latinx Black or Hispanic/Latinx and did not select “transgender”).

#### Substance use.

Past 30-day and lifetime substance use was queried with the following questions: “During the past 30 days, on how many days did you use? 0, 1, 2, 3—9, 10—19, or 20—days” and “During your life, how many times have you used the following substances?: 0, 1, 2, 3, 4—6, 7 or more times.” Past 30-day and lifetime use was assessed for each of the following substances: cigarettes, electronic cigarettes/e-cigarettes/other vaping device, marijuana, and at least one drink of alcohol. Responses to the past 30-day use question were categorized as 0 days or 1 or more days, and responses to the lifetime 30-day use questions were categorized as never or 1 or more times.

#### School-based stressors.

A 9-item measure assessed school-based victimization; an example item is “During the past 12 months, how many times on school property have you been made fun of because of your looks or the way you talk?” Item responses were dichotomized to 0 times or 1 or more times, and a continuous count variable summing the 9 items was created with a range of 0—9 with 9 as the highest level of victimization. [25] Experiences of harassment were assessed with questions focused on frequency of three types of harassment due to perceived or real belonging to specific social groups. Youth were asked, “During the past ***12 months,*** how many times on school property were you harassed or bullied for any of the following reasons? Your race, ethnicity, or national origin; Your gender (male or female); Because you are gay or lesbian or someone thought you were” which correspond to race-based, gender-based, and sexuality-based harassment, respectively. Responses for each harassment type were dichotomized to 0 times and 1 or more times.

#### Covariates.

Sociodemographic covariates included measures of reported sex, grade, and receipt of free or reduced school lunch. Reported sex was measured by the question, “What is your sex?” (male or female). Notably, the survey did not provide definitions of gender identity, sex, or designated sex at birth. For receipt of free or reduced school lunch, students were asked “Do you receive free or reduced-price lunches at school?” (no, yes, or I do not know).

#### Analytical plan

Descriptive and multivariable analyses were performed using STATA 16.1 (StataCorp, College Station, Texas) and the sampling plan and weights provided by WestEd using the STATA svy package. Descriptive analyses were conducted for demographics, substance use, and school-based stressors. Given the sample was weighted, estimated means were calculated for continuous variables and estimated prevalence was calculated for dichotomous variables. Logistic regression analyses were conducted to compare substance use (past 30-day and lifetime) and dichotomous school-based stressors between trans BLY and trans WY and between trans BLY and cis BLY and to examine associations between substance use and school-based stressors for trans BLY. Linear regression analyses were used to compare peer groups on school-based victimization, which is continuous. All regression analyses included grade, reported sex, and receipt of free or reduced school lunch (proxy for families’ socioeconomic status) as covariates given their likely associations with substance use [26,27]. For the past 30-day and lifetime use of each substance and school-based stressors, 4%–7% of data for each variable were missing. For linear and logistic regression analyses, inverse weights were used for missingness adjusting for grade, sex, receipt of free lunch, and derived peer group. All hypothesis testing used *p* < .05 significance level and 95% confidence intervals (CIs).

## Results

Data from 19,780 students were used for the analytical sample of this study because they were in 9th or 11th grade and belonged to derived peer groups of trans BLY, trans WY, or cis BLY. Table 1 presents the demographics by peer group. Approximately half of the analytic sample was in ninth grade (51%, weighted, 95% CI 50–52) and reported female sex (50%, weighted, 95% CI 49–51). The sample included 252 trans BLY (1.3%, weighted, 95% CI 1.1–1.5), 19,424 cis BLY (98.0%, weighted, 95% CI 97.8–98.2), and 104 trans WY (0.7%, weighted, 95% CI 0.6–0.8).

Table 2 provides prevalence estimates for 30-day and lifetime use of cigarettes, e-cigarettes/vaping, marijuana, and alcohol for each peer group. Table 3 provides prevalence estimates for all forms of harassment by peer group. For trans BLY, estimated prevalence of race-based, gender-based, and sexuality-based harassment were 33% (95% CI 27–39), 39% (95% CI 33–46), and 36% (95% CI 30–42), respectively. Table 3 also shows estimated means for each peer group for the continuous victimization variable; higher scores indicate higher victimization.

Prevalence of past 30-day and lifetime substance use for cigarettes, e-cigarette/vaping, marijuana, and alcohol were compared between trans BLY and trans WY and between trans BLY and cis BLY (Table 4). After adjusting for sociodemographic factors, there were no significant differences in prevalence of past 30-day or lifetime use of each substance when comparing trans BLY and trans WY. Trans BLY also had similar adjusted odds of all forms of harassment and similar levels of victimization compared to trans WY. However, trans BLY had higher adjusted odds of 30-day and lifetime use of each substance when compared with cis BLY. For example, for 30-day substance use, trans BLY had higher adjusted odds for use of each substance compared to cis BLY: cigarette (adjusted odds ratio [aOR] = 3.9, 95% CI 2.5–6.1), e-cigarette/vaping (aOR = 2.5, 95% CI 1.8–3.6), marijuana (aOR = 2.1, 95% CI 1.5–2.9), and alcohol (aOR = 1.9, 95% CI 1.4–2.6). Trans BLY also had higher adjusted odds of each form of harassment when compared to cis BLY: race-based harassment (aOR = 3.4, 95% CI: 2.6–4.6), gender-based harassment (aOR = 12.1, 95% CI 8.9–16.5), and sexuality-based harassment (aOR = 8.3, 95% CI: 6.3–11.0). Trans BLY had a higher level of victimization compared to cis BLY with an adjusted linear coefficient of 1.9 (95% CI: 1.4–2.3).

The multivariable logistic regression models assessing associations between substance use and school-based stressors for trans BLY are shown in Table 5. Race-based harassment and gender-based harassment were associated with higher odds of 30-day use and lifetime use of all substances. For example, experiencing race-based harassment was associated with higher odds of 30-day substance use: cigarette use (aOR = 5.3, 95% CI 2.1–13.7), vaping/e-cigarette use (aOR = 4.2, 95% CI 2.0–8.8), marijuana use (aOR = 3.3, 95% CI 1.7–6.5), and alcohol use (aOR = 3.9, 95% CI 2.0–7.7). Sexuality-based harassment was associated with higher odds of 30-day and lifetime use of marijuana and alcohol. Experiencing higher levels of victimization was associated with higher adjusted odds of 30-day and lifetime substance use of all substances.

## Discussion

To our knowledge, this is the first study investigating differences in substance use and associated school-based stressors among trans BLY and their peers. Using a large school- and population-based sample that included transgender youth allowed us to document disparities in substance use among trans BLY—youth that experience being gender diverse and minoritized based on race and ethnicity. To examine disparities in substance use, we compared trans BLY to trans WY and to cis BLY. As expected, we found substance use disparities experienced by trans BLY, and trans BLY’s substance use was associated with school-based victimization and harassment.

Counter to our hypothesis, trans BLY had similar odds of past 30-day and lifetime use of all substances queried compared to trans WY. Moreover, these two peer groups had similar odds of experiencing each form of harassment and similar levels of victimization. Consistent with our hypothesis, when compared to cis BLY, trans BLY experienced higher odds of past 30-day and lifetime use of each substance, race-based harassment, sexuality-based harassment, and higher levels of victimization. The comparisons between trans BLY and cis BLY are consistent with findings that transgender youth are at higher risk of substance use compared to cisgender peers [10,11,28]. Similarities in substance use that trans BLY have with trans WY and differences they have with cis BLY potentially point to the complex interplay of trans BLY’s unique minoritized experiences that may be associated with substance use. For example, a trans BLY who is perceived as different because of gender identity, race, and ethnicity may experience more stress, alienation, or loneliness leading to substance use as a coping mechanism or to fit in with peers.

In this study, school-based stressors were associated with substance use among trans BLY. These findings support the gender minority stress framework, which posits that minority related stressors are associated with poor health behaviors, specifically substance use, among gender-diverse individuals [19,28,29]. Additionally, our findings are consistent with the literature focused broadly on transgender youth [10]. Studies of sexual minority and gender-diverse youth suggest that minority-related stressors, including victimization, are strong predictors of substance use [10,23]. Interestingly, in our study, gender-based harassment and race-based harassment were both associated with use of all substances (cigarette, vaping/e-cigarette, marijuana, and alcohol) while sexuality-based harassment was only associated with use of marijuana and alcohol. Gender, including gender expression, and race are both outward characteristics while sexuality may or may not be outwardly expressed. Potentially, harassment targeting outward-facing characteristics or expression may lead to use of more substances to cope with such stressors or to fit in with peers.

In our study, trans BLY reported high rates of substance use, consistent with other population-based studies focused on transgender youth [10,11]. Our findings highlight the importance of further examining this public health issue and suggest the need for pediatric clinicians to screen transgender youth for substance use given their risk. Our study also identified modifiable risk factors—various forms of school-based harassment and victimization—that clinicians can identify and work with school-based mental health and counseling services to prevent and mitigate substance use among this population. Additionally, our findings can inform the development of peer interventions to prevent and intervene upon racism and transphobia in the school setting.

Trans BLY is a heterogenous group with diverse cultural experiences, and our study’s analytic approach of studying Black and Latinx transgender youth as a group is a crucial first step in examining shared experiences of having minoritized racial, ethnic, and gender identities. Moreover, this approach mirrors studies focused on Black and Latinx transgender adults that show disproportionately poorer health and psychosocial outcomes [30–32]. Further studies are needed to parse out mechanisms for the disparities and risk factors for substance use among trans BLY. Additional studies are needed to tease apart nuanced differences among specific minoritized groups and forms of stigma that may influence their substance use. As existing datasets may not include measures of racism or cultural factors, studying multiply minoritized youth with both qualitative and quantitative methods is critical to further elucidate their experiences and to target substance use with culturally informed interventions. Notably, as a group, the majority of trans BLY did not report substance use. Future studies to identify resilience factors that prevent substance use among these youth—for example, spirituality, cultural pride, and social connectedness—are potentially important measures for better understanding factors that protect youth against substance use. Since these measures were not included in the dataset, we were unable to examine them in the context of this research. Future research would do well to include such measures. Finally, the survey included distal social stressors in the form of victimization and harassment, but further studies are needed to explore proximal stress factors, such as internalized transphobia, and their impact on substance use.

Given the study’s observational design, we cannot infer causality among peer groups, substance use, and risk factors. Our study’s ability to detect differences between trans BLY and trans WY may be limited by modest sample sizes. Moreover, our study focused on past 30-day and lifetime substance use; further studies to assess the prevalence, frequency, and severity of substance use among trans BLY will be critical. Another limitation of our study is the potential for misclassification bias. Future studies should query gender identity separately from sexual identity. Also, sex was not defined or distinguished from gender identity; future studies should utilize current recommendations for identifying gender-diverse populations—that is, the “two-step approach” of querying sex assigned at birth and gender identity separately [33,34]. “Transgender’ was the only gender-diverse identity option in the survey; future studies should provide additional options for youth to identify as gender nonbinary, gender fluid, or genderqueer. Also, gender-based harassment was examined as a school-based stressor associated with substance use; this survey item assessed harassment due to male or female gender and not specifically due to gender identity or being gender diverse. Future studies should include items to specifically assess transphobia-based harassment. Furthermore, only students who attended school participated in the survey, so the study findings may not be generalizable to students not attending school; of note, data regarding the percentage of students in attendance during the period of data collection are not available. There is also a potential for attribution bias as youth were asked about perceived harassment due to various aspects of their identity—race, gender, and sexuality—as well as the potential for reporting and recall bias due to social desirability [35].

Strengths of the study include utilization of a large probability sample that is representative of the California high school student population, which allowed comparisons among peer groups. Moreover, this survey was administered in the school setting, an important environment for youth. School is also a potential setting for culturally informed interventions to support trans BLY and reduce their risk of substance use. The presence of student clubs, such as Gay-Straight Alliances, is associated with reduced risk of substance use and attenuating other health risks among gender-diverse and sexual minority students [36,37]. The Gay, Lesbian, and Straight Education Network advocates intersectional approaches to foster support for Black and Latinx gender-diverse and sexual minority youth [38,39], specifically to reduce school-based victimization and harassment, which our study shows is associated with substance use. Finally, our study demonstrates the relative vulnerability for substance use among trans BLY, reinforcing the need for schools, community-based organizations, and clinicians to work together to prevent substance use by addressing factors that increase risk of substance use with culturally informed interventions.

## Figures and Tables

**Table 1:** Demographics for Black and Latinx transgender youth and their peers

	Black and Latinx transgender youth (N = 252)	White transgender youth (N = 104)	Black and Latinx cisgender youth (N = 19,424)
	Prevalence (CI)^a^	Prevalence (CI)^a^	Prevalence (CI)^a^
Race/ethnicity^b^			
NH White	-	100%	-
NH Black	11% (7–18)	-	10% (9–10)
Hispanic/Latinx	89% (83–93)	-	90% (90–91)
Reported sex			
Female	65% (58–70)	61% (51–70)	50% (49–51)
Male	35% (30–42)	39% (30–49)	50% (49–51)
Grade			
9th	52% (46–58)	50% (40–59)	51% (50–52)
11th	48% (42–54)	50% (40–60)	49% (48–50)
Free or reduced lunch	59% (52–65)	17% (11–25)	68% (67–68)

CI = 95% confidence interval; NH = non-Hispanic/Latinx.

a Estimated prevalence with 95% confidence intervals is presented given weighted sample analyzed.

b Mutually exclusive categories of race and ethnicity were created.

**Table 2:** Estimated prevalence of substance use by peer group

	Black and Latinx transgender youth	White transgender youth	Black and Latinx cisgender youth
	Prevalence (CI)^a^	Prevalence (CI)^a^	Prevalence (CI)^a^
30-Day cigarette use	13% (9–18)	13% (8–21)	3% (3–4)
30-Day vaping	19% (l5–25)	25% (18–35)	9% (8–9)
30-Day marijuana use	27% (22–33)	22% (15–31)	14% (14–15)
30-Day alcohol use	29% (23–35)	32% (24–42)	19% (18–19)
Lifetime cigarette use	23% (18–30)	27% (19–36)	10% (9–10)
Lifetime vaping	39% (33–45)	39% (30–49)	30% (30–31)
Lifetime marijuana use	42% (35–48)	34% (25–43)	28% (27–28)
Lifetime alcohol use	48% (41–54)	47% (38–57)	36% (36–37)

CI = 95% confidence interval.

a Estimated prevalence for dichotomous measures with 95% confidence intervals is presented given weighted sample analyzed.

**Table 3:** Estimated prevalence and means of school-based stressors by peer group

Dichotomous measures	Black and Latinx transgender youth	White transgender youth	Black and Latinx cisgender youth
	Prevalence (CI)^a^	Prevalence (CI)^a^	Prevalence (CI)^a^
Race-based harassment	33% (27–39)	25% (18–33)	13% (12–13)
Gender-based harassment	39% (33–46)	39% (30–48)	6% (6–7)
Sexuality-based harassment	36% (30–42)	45% (35–54)	7% (6–7)
Continuous measure	Black and Latinx transgender youth	White transgender youth	Black and Latinx cisgender youth
	Mean (CI)^a^	Mean (CI)^a^	Mean (CI)^a^

Victimization (range 0–9)^b^	3.4 (3.0–3.9)	3.0 (2.4–3.6)	1.7 (1.6–1.7)

CI = 95% confidence interval.

a Given the weighted sample, estimated prevalence for dichotomous measures with 95% confidence intervals and estimated means for continuous measures with 95% confidence intervals are presented.

b Higher victimization scores correspond with higher levels of victimization.

**Table 4:** Substance use and school-based stressors comparing Black and Latinx transgender youth to peer groups with adjusted odds ratios and regression coefficients

Dichotomous measures^a^	White transgender youth as the referent group	Black and Latinx cisgender youth as the referent group
	AOR (95% CI)	AOR (95% CI)
30-Day cigarette	1.0 (0.5–2.0)	3.9 (2.5–6.1)^b^
30-Day vaping	0.7 (0.4–1.2)	2.5 (1.8–3.6)^b^
30-Day marijuana	1.2 (0.7–2.1)	2.1 (1.5–2.9)^b^
30-Day alcohol	0.9 (0.5–1.5)	1.9 (1.4–2.6)^b^
Lifetime cigarette	0.7 (0.4–1.3)	2.5 (1.8–3.6)^b^
Lifetime vaping	0.8 (0.5–1.4)	1.4 (1.1–1.9)^b^
Lifetime marijuana	1.3 (0.7–2.1)	1.8 (1.4–2.5)^b^
Lifetime alcohol	0.9 (0.5–1.5)	1.6 (1.2–2.1)^b^
Race-based harassment	1.4 (0.8–2.4)	3.4 (2.6–4.6)^b^
Gender-based harassment	1.2 (0.7–2.2)	12.1 (8.9–16.5)^b^
Sexuality-based harassment	0.7 (0.4–1.1)	8.3 (6.3–11.0)^b^
Continuous measure^c^	White transgender youth as the referent group	Black and Latinx cisgender youth as the referent group
	Adjusted linear regression coefficient (95% CI)	Adjusted linear regression coefficient (95% CI)

Victimization	0.4 (−0.3 to 1.2)	1.9 (1.4–2.3)^d^

AOR = adjusted odds ratio; CI = 95% confidence interval.

a Logistic regression analyses were conducted for dichotomous measures and were adjusted for grade, reported sex, and receiving free or reduced lunch. Adjusted odds ratios are presented with 95% confidence intervals.

b *p* < .05 as indicated by 95% confidence interval not crossing 1.

c Linear regression analyses were conducted for continuous measures and were adjusted for grade, reported sex, and receiving free or reduced lunch. Regression coefficients are presented with 95% confidence intervals.

d *p* < .05 as indicated by 95% confidence interval not crossing 0.

**Table 5:** Stressors related to minoritized identities as predictors of substance use in Black and Latinx transgender youth with adjusted odds ratios

	Race-based harassment	Gender-based harassment	Sexuality-based harassment	Victimization
30-Day cigarette use	5.3 (2.1–13.7)^a^	5.4 (1.8–16.0^a^	2.6 (1.0–6.7)	1.3 (1.1–1.5)^a^
30-Day vaping	4.2 (2.0–8.8)^a^	2.8 (1.3–6.0)^a^	2.0 (1.0–4.3)	1.3 (1.1–1.4)^a^
30-Day marijuana use	3.3 (1.7–6.5)^a^	5.6 (2.6–12.1)^a^	3.7 (1.9–7.4)^a^	1.3 (1.2–1.5)^a^
30-Day alcohol use	3.9 (2.0–7.7)^a^	4.2 (2.1–8.3)^a^	2.8 (1.5–5.4)^a^	1.3 (1.1–1.4)^a^
Lifetime cigarette use	3.7 (1.8–7.6)^a^	3.4 (1.6–7.0)^a^	2.0 (1.0–4.1)	1.2 (1.1–1.4)^a^
Lifetime vaping	2.5 (1.3–4.8)^a^	2.1 (1.1–3.8)^a^	1.3 (0.7–2.4)	1.2 (1.1–1.3)^a^
Lifetime marijuana use	3.9 (2.0–7.7)^a^	4.3 (2.2–8.2)^a^	2.5 (1.4–4.7)^a^	1.3 (1.1–1.4)^a^
Lifetime alcohol	2.7 (1.4–5.1)^a^	3.6 (2.0–6.6)^a^	2.5 (1.4–4.4)^a^	1.3 (1.1–1.4)^a^

Logistic regression analyses were conducted and adjusted for grade, reported sex, and receiving free/reduced lunch. Adjusted odds ratios are presented with 95% confidence intervals.

a *p* < .05 as indicated by 95% confidence interval not crossing 1.
